# Supplementary material for: A modified BPaL regimen for tuberculosis treatment replaces linezolid with inhaled spectinamides
Source: eLife. 2024 Oct 8;13:RP96190. doi: 10.7554/eLife.96190 (PMC11460978; doi:10.7554/eLife.96190)
Supplement: Supplementary file 1. [file elife-96190-supp1.docx]

**Supplementary Data**

**A Modified BPaL Regimen for Tuberculosis Treatment replaces Linezolid with Inhaled Spectinamides**

Malik Zohaib Ali ^1,2,6^, Taru S. Dutt ^1,2^, Amy MacNeill ^2^, Amanda Walz ^1,2^, Camron Pearce ^1,2.6^, Ha Lam^1,2^, Jamie Philp ^1,2^, Johnathan Patterson ^1,2^, Marcela Henao-Tamayo ^2^, Richard E. Lee ^3^, Jiuyu Liu ^3^, Gregory T. Robertson ^1,2^, Anthony J. Hickey ^4^, Bernd Meibohm ^5^, Mercedes Gonzalez-Juarrero ^1,2^

^1^Mycobacteria Research Laboratories, Colorado State University, 80523, USA

^2^Microbiology, Immunology and Pathology, Colorado State University, 80523, USA

^3^Department of Chemical Biology and Therapeutics, St. Jude Children’s Research Hospital, 38105, USA

^4^Technology Advancement and Commercialization, RTI International, 27709, USA

^5^Department of Pharmaceutical Sciences, University of Tennessee Health Science Center, 38163, USA

^6^Program in Cell & Molecular Biology, Colorado State University, 80523, USA

**Supplementary File 1**

| **Antibody** | **Specie** | **Type** | **Company** | **Catalogue #** | **Concentration** | **pH** | **Opal** |
| --- | --- | --- | --- | --- | --- | --- | --- |
| CD8 | Rabbit | mAb | CST | D4W2Z | 1:400 | 6 | 480 |
| CD4 | Rat | mAb | Thermo Fisher | 4SM95 | 1:200 | 6 | 520 |
| B220 | Rat | mAb | BD Pharm | RA3-6B2 | 1:500 | 6 | 570 |
| FoxP3 | Rabbit | mAb | R&D | MAB8214 | 1:200 | 6 | 620 |
| Ly6G | Rabbit | mAb | CST | 87048 | 1:100 | 6 | 690 |
| F4/80 | Rabbit | mAb | CST | D4C8V | 1:100 | 6 | 780 |
